# Gel-immersion peroral cholangioscopy facilitates targeted sampling of a liver abscess

**DOI:** 10.1055/a-2967-8096

**Published:** 2026-10-01

**Authors:** Masahiro Yamamura, Takeshi Ogura, Saori Ueno, Nobu Nishioka, Takafumi Kanadani, Hiroki Nishikawa

**Affiliations:** 1 Pancreatobiliary Advanced Medical Center 38588 Osaka Medical and Pharmaceutical University HospitalTakatsukiOsakaJapan; 2 2nd Department of Internal Medicine 13010 Osaka Medical and Pharmaceutical UniversityTakatsukiOsakaJapan; 3 Endoscopy Center 38588 Osaka Medical and Pharmaceutical University HospitalTakatsukiOsakaJapan

Peroral cholangioscopy (POCS) enables direct visualization and targeted sampling within the bile ducts. When a liver abscess communicates with an intrahepatic bile duct, POCS can provide direct access to the abscess cavity. However, bile, pus, and necrotic debris may decrease visibility, making targeted sampling challenging. Water irrigation often fails to provide a stable visual field because water rapidly mixes with bile and purulent materials. Gel-immersion endoscopy using a transparent viscous gel (Viscoclear; Otsuka Pharmaceutical Factory, Tokushima, Japan) has been reported to provide a stable endoscopic view. 1​ 2​ 3​ 4​ ​5​ We present gel-immersion POCS-guided visualization and sampling of a liver abscess through an intrahepatic bile duct ( Video 1 ).

**Video 1** Gel-immersion peroral cholangioscopy provided clear visualization of a liver abscess communicating with an intrahepatic bile duct, enabling targeted biopsy, culture sampling, guidewire placement, and selective drainage.


A 70-year-old woman with a previously placed self-expandable metal stent (SEMS) for malignant biliary obstruction presented with fever during systemic chemotherapy. Computed tomography (CT) revealed multiple liver abscesses ( Fig. 1 ). Endoscopic retrograde cholangiopancreatography (ERCP) demonstrated an abscess cavity communicating with an intrahepatic bile duct ( Fig. 2 ). The previously placed SEMS was removed and replaced with transpapillary stents: a SEMS in the common bile duct and plastic stents in both hepatic ducts. However, fever and inflammation persisted, and follow-up CT showed the progression of the abscesses. During repeat ERCP, a cholangioscope was advanced into an abscess cavity ( Fig. 3 ). Water immersion failed to provide a clear view because of bile and purulent materials. Gel injection provided a clear and stable endoscopic view, enabling safe targeted biopsy and specimen collection for culture ( Fig. 4 ). Finally, two plastic stents were placed in the left-sided abscess cavities, one in another abscess cavity, and one in the anterior sectoral duct ( Fig. 5 ). Antibiotics were modified according to the culture results, and the patient condition improved and was discharged.

**Fig. 1:**
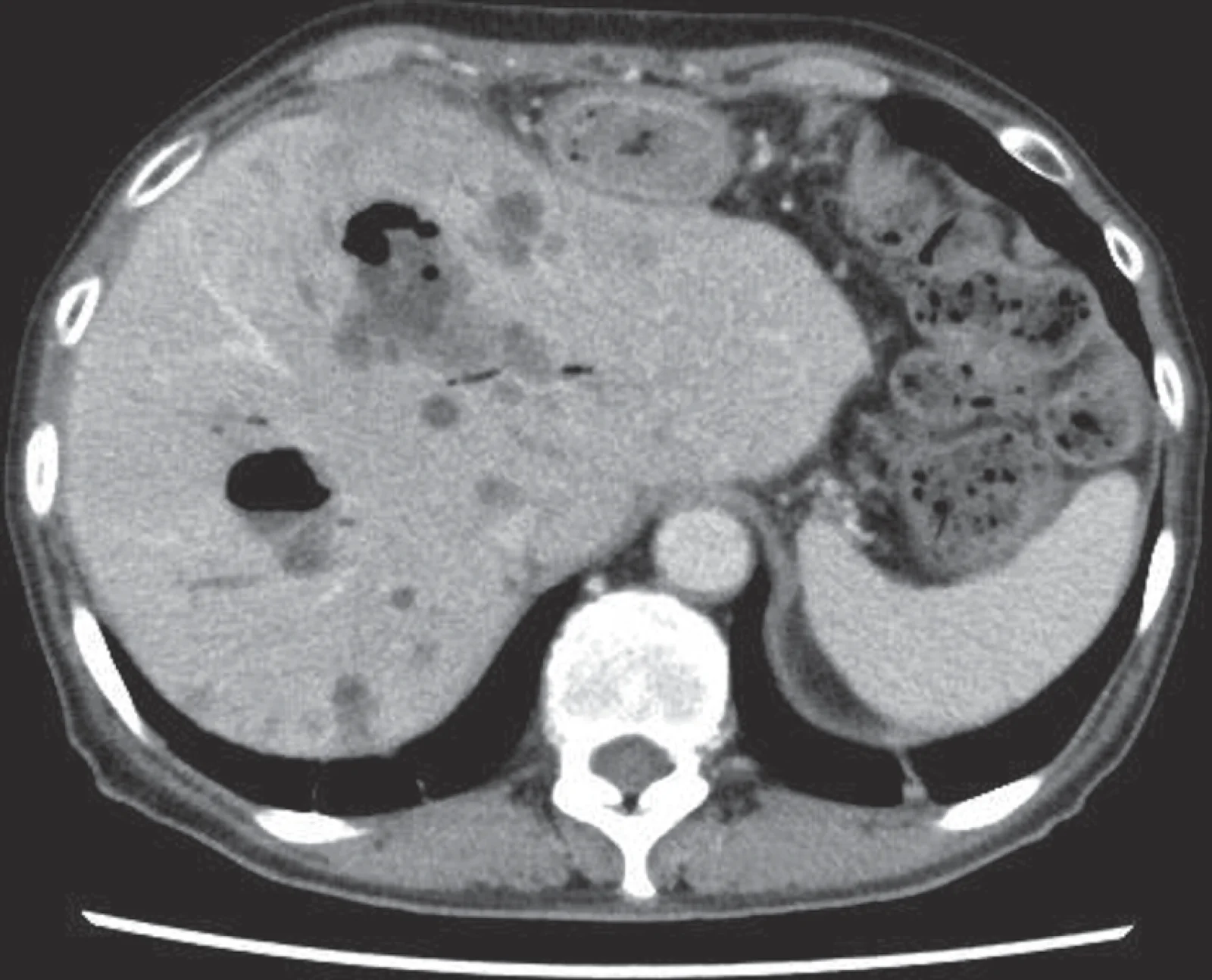
Computed tomography (CT) revealed multiple liver abscesses.

**Fig. 2:**
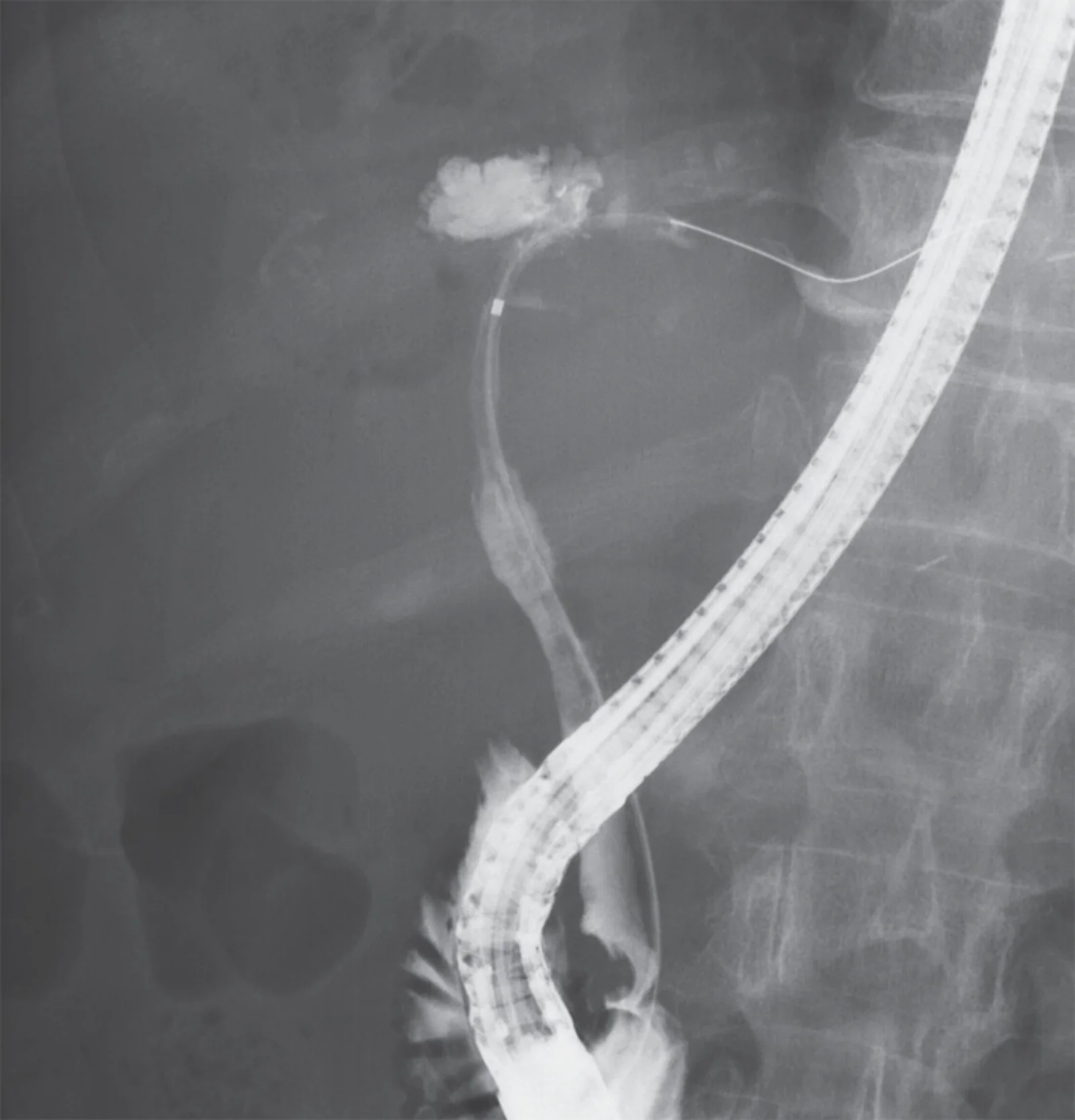
Cholangiography revealed an abscess cavity communicating with an intrahepatic bile duct.

**Fig. 3:**
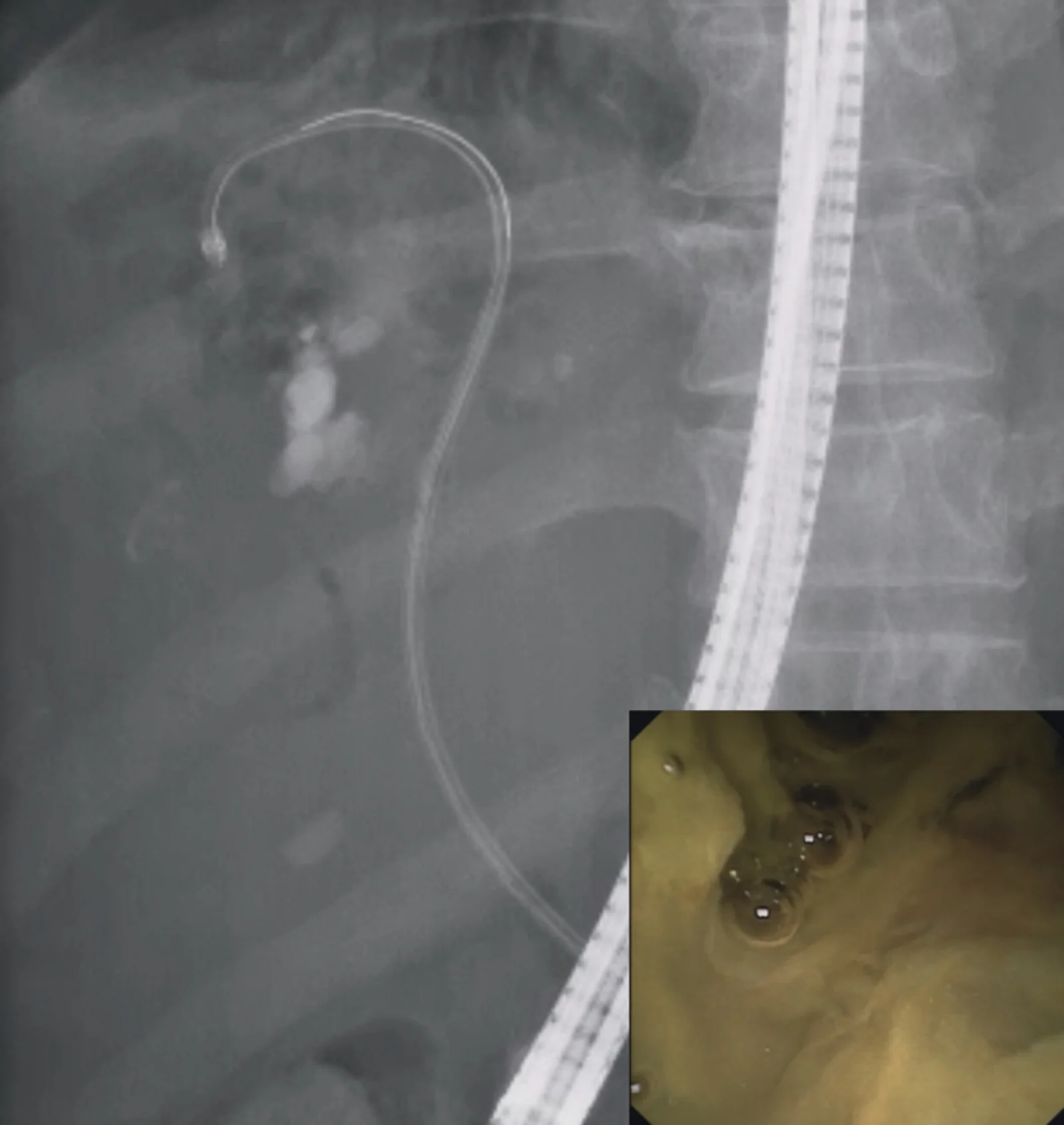
A cholangioscope was advanced into an abscess cavity.

**Fig. 4:**
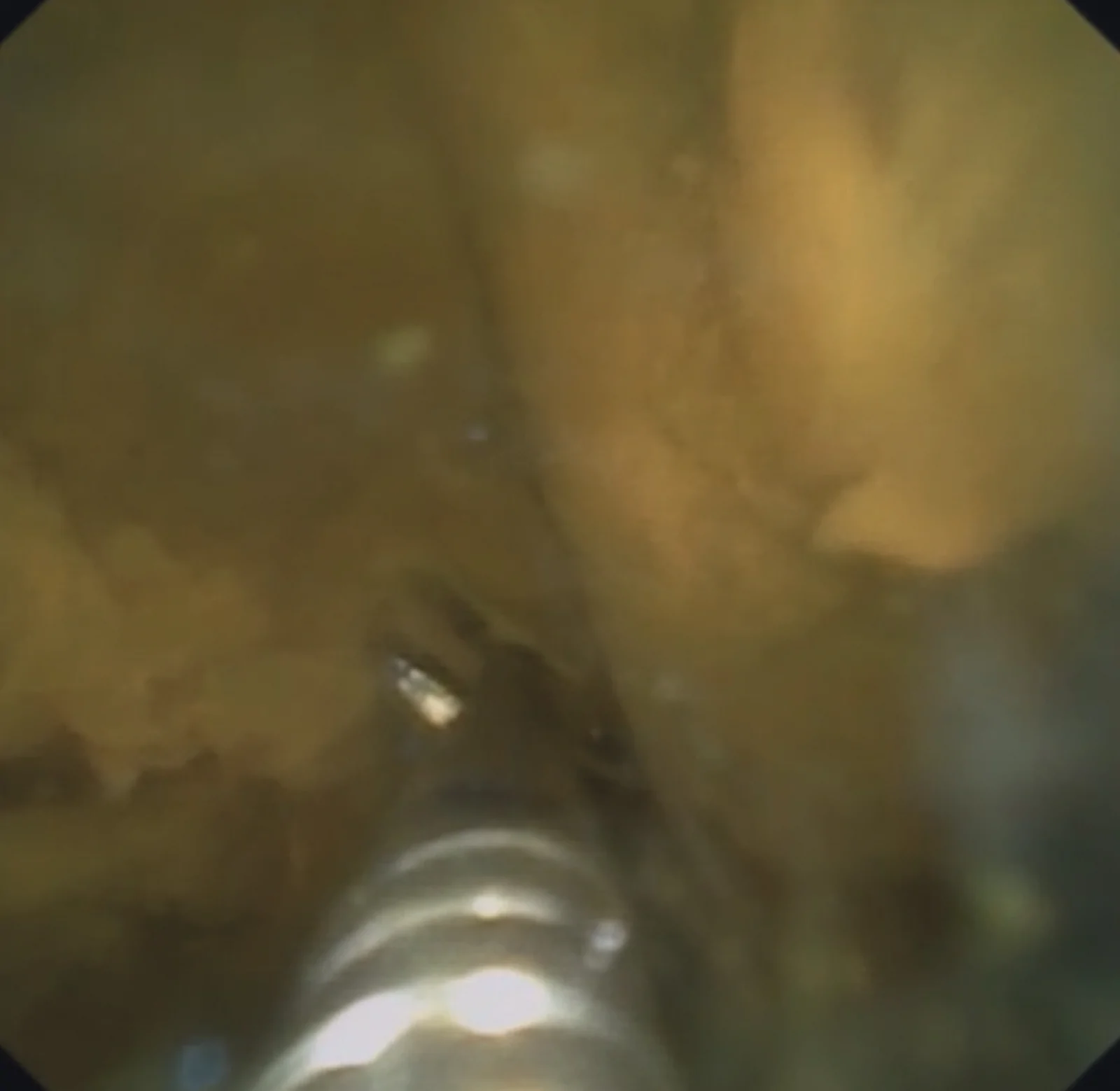
Targeted biopsy and specimen collection for microbiological culture under gel-immersion cholangioscopic guidance.

**Fig. 5:**
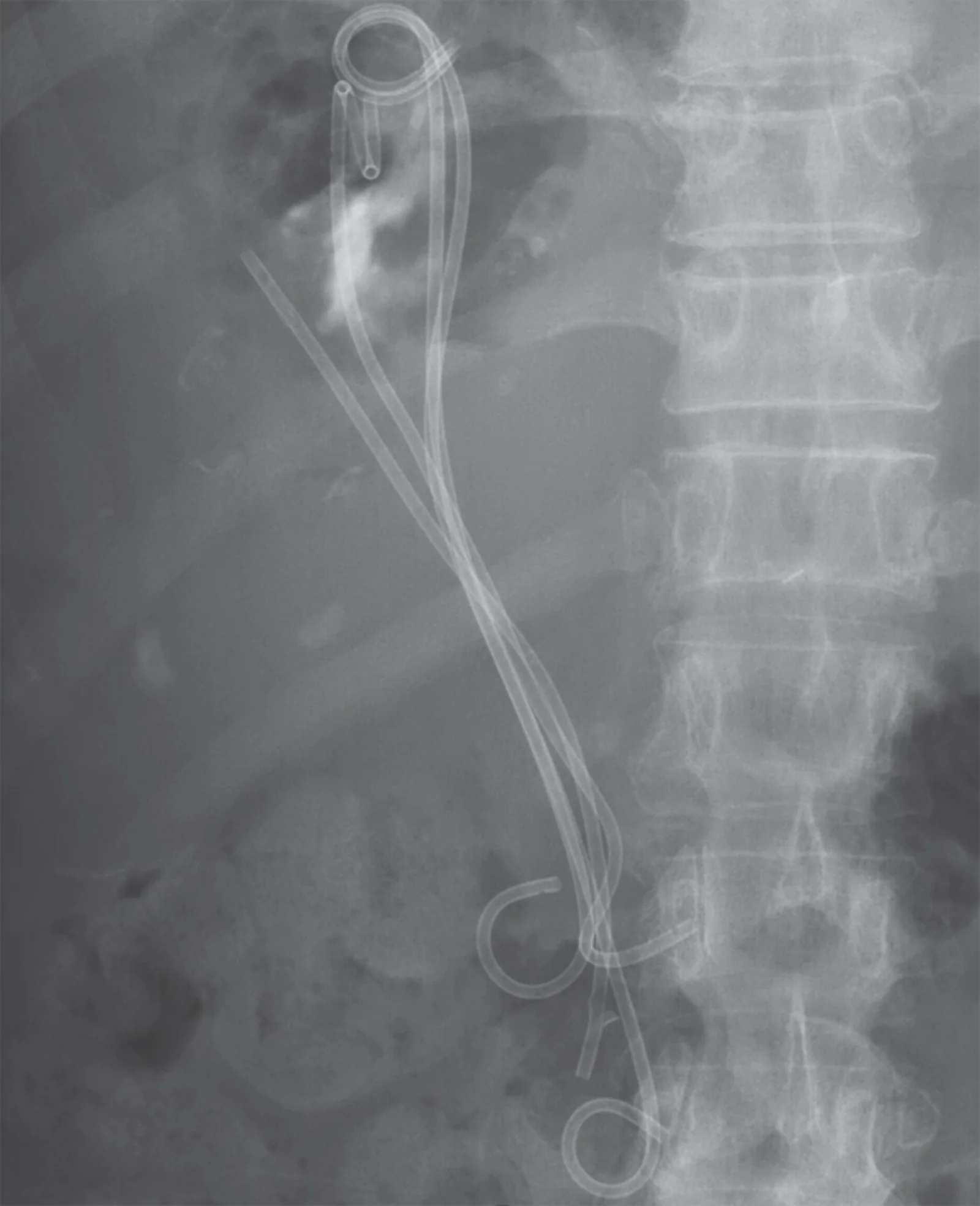
Two plastic stents were placed in the left-sided abscess cavities, one in another abscess cavity and one in the anterior sectoral duct.

Gel-immersion POCS may facilitate targeted sampling and selective drainage of liver abscesses communicating with the bile ducts.

Endoscopy_UCTN_Code_TTT_1AR_2AL

## Declaration of GenAI Use

During the preparation of this manuscript, the authors used ChatGPT (OpenAI) for English translation and language editing. The authors reviewed and edited the output and take full responsibility for the content of the manuscript.
